# Is culture the key? Emotional intelligence, autonomous motivation and physical activity of student-athletes from China and Russia

**DOI:** 10.3389/fpsyg.2024.1420430

**Published:** 2024-07-10

**Authors:** Liudmila Nikolaevna Rogaleva, Tao Zhong, Alexandre Garcia-Mas

**Affiliations:** ^1^Institute of Physical Culture, Sports and Youth Policy, Ural Federal University, Yekaterinburg, Russia; ^2^College of Sport and Health, Henan Normal University, Xinxiang, China; ^3^Department of Psychology, University of the Balearic Islands, Palma, Spain

**Keywords:** emotional intelligence, motivation, physical activity, student-athletes, culture

## Abstract

Emotional intelligence is considered as an important factor impacting on sports motivation of students-athletes. Meanwhile the role of culture in the development of emotional intelligence is still insufficiently studied in sports psychology. The purpose of the study included comparing the indicators of emotional intelligence of student-athletes in China and Russia, identifying the relationship between emotional intelligence, sports motivation and physical activity, as well as studying the prognostic effect of emotional intelligence on autonomous motivation. The research was done among 474 student- athletes. In Chinese students sample (*N* = 281), the 163 men and 118 women. In the Russian student sample (*N* = 193), there were 64 men and 129 women. The following research methods were used: emotional intelligence scale, the sport motivation scale-6, the international physical activity questionnaire. The results of the study have showed that the level of emotional intelligence of Chinese student-athletes is higher than that of Russian students-athletes. Positive correlations between emotional intelligence, autonomous motivation and physical activity were found in both samples. At the same time, the correlation coefficient in the Russian sample was lower. A predictive relationship between emotional intelligence and autonomous motivation has been established; meanwhile the self-assessment of emotions and emotion regulation make the greatest contribution to autonomous motivation in the Chinese sample, while in the Russian sample there is only the use of emotions. Considering the cultural context can contribute to the preparation effective programs for the development of emotional intelligence and autonomous motivation for physical activity.

## Introduction

Sports motivation is one of the main reasons when students choose sports specialties. Therefore, studying the sport motivation of Chinese and Russian student-athletes and its determining factors is important for understanding long-term commitment to sports and physical activity. Past research on sport motivation has been conducted either on non-sport students (Deng et al., 2023) or on student-athletes in the context of the dual-career theory (Stambulova et al., 2015). In the first and second case, the authors do not consider the differences between Eastern and Western culture and its influence on athletic motivation.

At the same time, Pan and Huang (2019) comparing Western and Eastern culture note that Western culture is focused on the conquest of nature, so sports are oriented toward competition and individual achievement, while Eastern culture is focused on ethics, so Eastern sports culture is characterized by a focus on the development of “mind and spirit.” In this context, Chinese student-athletes and Russian student-athletes belong to different sports cultures. Traditional Chinese sports culture is based on the philosophical ideas of “harmony of man and nature,” preservation of health through the “unity of body and spirit,” and focus on psychophysical improvement (Song and Shen, 2021). Russian sports culture is closely related to the values and ideas of Western sports culture, with the desire for excellence, maximum development of physical qualities (Zagrevskaya, 2019). The study of the influence of culture on the motivation to engage in physical activity implies the analysis of the main directions of its research.

These studies are concentrated around three key areas. The first concerns the study of students' sports motivation in the context of socio-demographic characteristics (Ekelund et al., 2006), and the role of external and internal factors influencing it (Wang et al., 2018). The second is related to the substantiation of the relationship between different types of sports motivation and the level of physical activity (Liu et al., 2023). It should be noted that in recent years, most research on this problem has been carried out within the framework of the theory of self-determination, which considers three different motivational structures depending on the degree of self-determination: autonomous motivation, controlled motivation, and amotivation (Deci and Ryan, 2000). Autonomous motivation is considered as the most self-determined, since it is associated with the focus on performing activities, in accordance with the goals and motives of the personality. Autonomous motivation is associated with volitional effort made out of pleasure, personal importance, or in accordance with personal values (Ryan and Deci, 2020). In contrast, controlled motivation involves engaging in behavior driven by external factors such as potential reward, perceived approval from others, or avoidance of punishment or guilt. Amotivation refers to a state of lack of motivation and interest, and a person cannot see the exact reason for performing an action (Wong and Law, 2002).

Numerous studies have proven the effect of autonomous motivation on increased physical activity and commitment to sports (Vallerand, 2007). At the same time, it is important to note that researchers emphasize that internal motivation (pleasure and challenge) is more typical for students who play sports, in turn, for students focused on physical exercise, external motives (appearance, weight, and stress reduction) are more characteristic (Diehl et al., 2018). The third direction justifies strategies, pedagogical conditions, or interventions related to increasing sports motivation (Ntoumanis et al., 2021).

Thus, the main vector of the conducted research is aimed at studying the factors contributing to the increase of sports motivation, and mainly the autonomous motivation of students, which is revealed more among student-athletes. In recent years, more and more attention has been paid to emotional intelligence in the study of personal factors affecting sports motivation (Laborde et al., 2016; Sukys et al., 2019).

Emotional intelligence is understood as a fundamental ability that ensures the effective integration of cognitive and emotional resources that ensure the effectiveness of activities (Côté and Miners, 2006). The results of the conducted research on the role of emotional intelligence in sports are quite contradictory. Some studies prove that emotional intelligence plays a significant role in increasing sports motivation and physical activity, contributing to the growth of athletic performance (Castro-Sánchez et al., 2018), while other studies indicate a slight connection or lack of interrelationships between them (Mazhar et al., 2021). This indicates the complexity of studying the phenomenon of emotional intelligence, which depends on sports experience (Ubago-Jiménez et al., 2019), socio-demographic and individual characteristics (Laborde et al., 2016; Rodriguez-Romo et al., 2021), and on some cultural factors (Fernández-Berrocal et al., 2005; Miranda-Rochín et al., 2023).

All the above confirms the importance of studying autonomous motivation among students at physical education universities and its determining factors. At the same time, it should be noted that emotional intelligence plays a critically important role in the training of future sports teachers (Malinauskas and Vazne, 2014), as it ensures their favorable social development, the ability to maintain both their internal motivation and the internal motivation of future students (Mercader-Rubio et al., 2023).

The conducted research has revealed that among student-athletes studying in sports specialties, there is a relationship between three dimensions of emotional intelligence (emotional attention, emotional clarity, and emotional regulation) and internal motivation (to acquire knowledge, to achieve goals and to search for incentives (Mercader-Rubio et al., 2023). At the same time, in these works the reasons for the differences in the emotional intelligence of student-athletes are not explained.

The answer to this question is to a certain extent provided by cross-cultural studies of the emotional intelligence of student-athletes. Quinaud et al. (2020), using the theory of building a dual career (Stambulova et al., 2015) when studying the motivation and identity of student-athletes from Brazilian and Portuguese universities, has revealed that student-athletes from Brazil are characterized by a higher level of sports motivation and pleasure from sports compared to Portuguese athletes- students. The obtained results indicate the influence of culture on the internal motivation and emotional intelligence of student-athletes.

Another cross-cultural study proved that Latvian student-athletes, unlike Lithuanian ones, have a better ability to use their own positive emotional experience (optimism) (Malinauskas and Vazne, 2014). Researchers have noted that the study of differences between the emotional intelligence of university students in Mexico and Spain is justified by the fact that emotional intelligence is mediated by cultural and individual variables (Miranda-Rochín et al., 2023).

Thus, the research results prove the important role of culture in the development of emotional intelligence of student–athletes, but they are not numerous, are limited by empirical data, and do not have sufficient theoretical justification. Therefore, in our opinion, in the context of studying the problem of the influence of emotional intelligence on the motivation of self-determination of student-athletes, the following questions remain unresolved: What is the role of culture in shaping the emotional intelligence of student–athletes? And to what extent can cultural differences in emotional intelligence influence the motivation of self-determination and physical activity of student-athletes? To answer these questions, we formulated the purpose of the study: to study the relationship between emotional intelligence, sports motivation and the level of physical activity of students at physical education universities in China and Russia.

The theoretical basis of the research is based on an integrative approach, including a cultural approach, the theory of emotional intelligence and the theory of motivation of self-determination. The cultural approach recognizes the existence of significant differences in the sports culture of the East and the West (Chen and Zhong, 2011; Song and Shen, 2021), as well as the influence of the culture of the East and the West on emotional self-regulation (Zhang and Cross, 2011).

**Figure 1:**
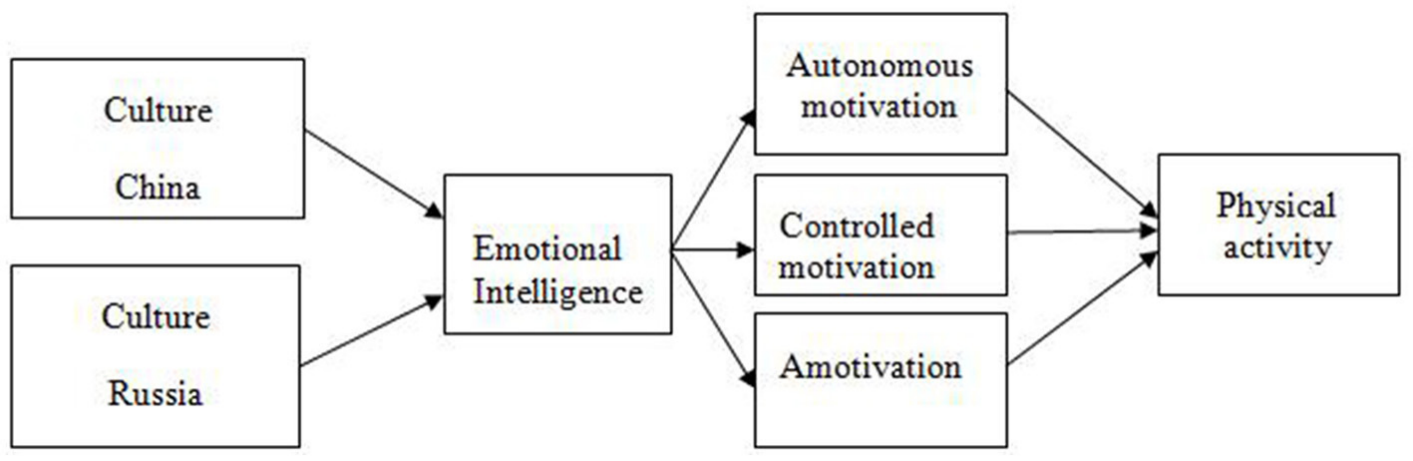
The theoretical model of the relationship between emotional intelligence, sports motivation (autonomous motivation, controlled motivation, and amotivation) and physical activity in the aspect of cultural differences.

Since the sports culture of the East and West can shape the way people think, act and regulate their emotions, our study suggests considering the influence of culture on emotional intelligence, which is considered as a predictor of autonomous motivation to engage student-athletes in physical activity. A theoretical is presented in Figure 1.

### General hypothesis

Our hypothesis is that China's sports culture is influenced by cultural traditions, which prioritize achieving inner harmony through psychophysical practices to manage one's emotions. As a result, student-athletes from China may have different levels of emotional intelligence compared to those from Russia.

### Particular hypotheses

Three hypotheses were tested during the study:

Hypothesis 1: Significant differences in the indicators of emotional intelligence in the group of Chinese and in the group of Russian student athletes will be determined.
Hypothesis 2: The existence of significant differences in the relationship between emotional intelligence, motivation and physical activity in a group of Chinese and a group of Russian student-athletes will be determined.
Hypothesis 3: The existence of significant differences in the influence of emotional intelligence and motivation in predicting behavior during physical activity in a group of Chinese and in a group of Russian student-athletes will be determined.

## Materials and methods

### Research design

The present study utilized a quantitative research design to address the research hypotheses (Figgou and Pavlopoulos, 2015). Research data from Chinese and Russian student-athletes were collected via a survey-based approach. Correlational models and regression models were adopted to test the effects among the study variables of emotional intelligence, sport motivation and physical activity.

### Participants

The research was done among 474 students-athletes from Chinese and Russian. Convenience sampling was used for participant recruitment. There were 281 Chinese students-athletes, including 163 men, average age 19.39 + 1.84 (from 17 to 23 years old) and 118 women average age 19.02 + 1.41 (from 17 to 22 years old). The group of Russian students-athletes included 193 participants, 64 men average age 19.5 ± 2.27 (from 17 to 25 years old) and 129 women average age 19.17 ± 2.19 (from 16 to 25 years old). All participants had experience in sport activity and were studying on programs related to sport. All participants gave their voluntary consent to participate in the research.

### Measures

#### Emotional intelligence

It was assessed using the emotional intelligence scale of Wong and Law (2002). The scale has been used internationally, demonstrating satisfactory validity and reliability for research purposes (Extremera Pacheco et al., 2019). The back-translation method was employed to translate the English version of the scale into both languages. Specifically, the instrument was translated into both languages by the bilingual authors. Subsequently, the translated versions were translated back into English by a different bilingual colleague in their institutions. The back-translated versions were then compared to the original instrument in order to identify and correct any differences, thus ensuring that they accurately reflected the original meaning.

#### Motivation

Students' motivation for sports activities was assessed using the Sports Motivation Scale-6 (SMS-6) (Mallett et al., 2007). The scale consists of 24 items, four items each, assessing intrinsic motivation, integrated regulation, identified regulation, introjected regulation, external regulation, and amotivation. Based on the level of internalization of motivation and consistent with past research, intrinsic motivation, integrated regulation, and identified regulation were grouped as autonomous motivation, whereas introjected regulation and external regulation were grouped as controlled motivation (Mouratidis et al., 2021). This instrument has been reported to have acceptable psychometric properties (Blecharz et al., 2015). The translation procedure was the same as employed in the previous scale.

The Cronbach's Alpha value gives an indication of the internal consistency and reliability of the data. Its range is 0–1, and its value should be >0.70 to be considered acceptable internal consistency. Emotional intelligence: the values of internal consistency (Cronbach's alpha), in the Chinese and Russian cultural contexts, were 0.946 and 0.849 for self-emotions appraisal, 0.939 and 0.800 for others-emotions appraisal, 0.943 and 0.780 for use of emotion, 0.948 and 0.872 for regulation of emotion, respectively. Motivation: the values of internal consistency (Cronbach's alpha), in the Chinese and Russian cultural contexts, were 0.952 and 0.927 for autonomous motivation, 0.882 and 0.852 for controlled motivation, and 0.846 and 0.731 for amotivation, respectively.

#### Physical activity

Physical activity levels were measured using the International Physical Activity Questionnaire Short Form (IPAQ-SF). The IPAQ-SF has been widely used and validated in international studies. Both the Chinese and Russian versions of the questionnaire were available for use. This measure was developed to facilitate international research on physical activity and has been widely used and accepted as a useful tool for measuring behavior (Ekelund et al., 2006).

### Procedure

The authors first created a web-based survey that utilized the tools used in the current study. After customizing the survey, the authors coordinated with their colleagues at their institutions to obtain permission to conduct the survey. Once permission was granted, a survey invitation containing information about the study and a QR code for our survey was distributed to college students.

Interested participants could access the survey by scanning the QR code of the survey. In the introduction to the survey, participants were informed that their participation in the study was anonymous and voluntary; they had the right to withdraw at any time if they did not wish to continue. Participants were also informed that by completing and returning the survey, they implied their consent to participate.

### Statistical analysis

The data were imported into SPSS version 26.0 for statistical analysis. Descriptive analysis was used to outline the bivariate correlations of the study variables, namely indicators of emotional intelligence, motivation, and physical activity. In line with the hypothesis, we then tested if there were any significant differences in scores of emotional intelligence across the Chinese and Russian student samples using independent samples *t-*test. Furthermore, we tested regression models in which indicators of emotional intelligence were set as independent variables, while autonomous motivation was set as a dependent variable. This was done to explore how emotional intelligence would affect autonomous motivation, a key factor in self-determination theory for human development. Additionally, we conducted regression analyses in both Chinese and Russian student participants to examine the role of emotional intelligence and motivation in predicting physical activity behavior.

## Results

To test hypothesis 1, we conducted a *t-*test of independent samples for differences in emotional intelligence dimensions on data from different countries. The results showed that Chinese students had higher scores on self-emotion appraisal (Chinese 5.43 ± 1.12 vs. Russian 5.10 ± 1.27, *p* < 0.01), emotional evaluation of others (Chinese 5, 32 ± 1.22 vs. Russian 5.08 ± 1.12, *p* < 0.05), emotion utilization (Chinese 5.32 ± 1.21 vs. Russian 5.00 ± 1.10, *p* < 0.01), and emotion regulation (Chinese 5.17 ± 1.32 vs. Russian 4.89 ± 1.30, *p* < 0.05). Thus, the statistical significance of differences in scores was established. Higher emotional intelligence scores prove the presence of cultural factor influence on the development of emotional intelligence, and as we hypothesized emotional intelligence is higher among Chinese student-athletes.

To test hypothesis 2, we conducted bivariate correlation analysis using Pearson correlation coefficient. Data of coefficients for Chinese and Russian samples are presented above and below the Table 1 diagonal line, respectively. According to the findings, in the sample of Chinese student-athletes, emotional intelligence indicators have higher correlation with autonomous motivation than with controlled motivation. The correlations between autonomous motivation and emotional intelligence indicators were as follows: self-emotion appraisal (r = 0.61, *p* < 0.01), other's emotion appraisal (r = 0.57, *p* < 0.01), use of emotion (r = 0.60, *p* < 0.01), and regulation of emotion (r = 0.563, *p* < 0.01). In addition, the correlation coefficients of emotional intelligence and amotivation indicators were insignificant, indicating that there was no relationship between them. In a sample of Chinese student-athletes, positive correlations were observed between emotional intelligence scores and physical activity. Similar results could be observed in the Russian sample but the relationship between emotional intelligence and autonomous motivation in the Chinese sample was higher than that in the Russian sample. At the same time the relationship between autonomous motivation and physical activity was similar in Chinese and Russian samples (r = 0.345, *p* < 0.01 and r = 0.359, *p* < 0.01 respectively).

**Table 1:** Bivariate correlation analyses results of study variables.

	**SEA**	**OEA**	**UOE**	**ROE**	**AUM**	**CNM**	**AM**	**PA**
SEA	-	0.815^**^	0.786^**^	0.668^**^	0.610^**^	0.479^**^	−0.063	0.333^**^
OEA	0.453^**^	-	0.773^**^	0.691^**^	0.570^**^	0.505^**^	−0.029	0.347^**^
UOE	0.601^**^	0.419^**^	-	0.786^**^	0.600^**^	0.511^**^	−0.018	0.402^**^
ROE	0.695^**^	0.338^**^	0.546^**^	-	0.563^**^	0.503^**^	0.001	0.374^**^
AUM	0.280^**^	0.282^**^	0.420^**^	0.239^**^	-	0.778^**^	0.064	0.345^**^
CNM	0.171^*^	0.202^**^	0.343^**^	0.177^*^	0.839^**^	-	0.352^**^	0.296^**^
AM	−0.180^*^	−0.013	−0.179^*^	−0.197^**^	0.029	0.207^**^	-	−0.073
PA	0.174^*^	0.130	0.327^**^	0.152^*^	0.359^**^	0.289^**^	0.006	-

SEA, self-emotion appraisal; OEA, other's emotion appraisal; UOE, use of emotion; ROE, regulation of emotion; AUM, autonomous motivation; CNM, controlled motivation; AM, amotivation; PA, physical activity; Correlation coefficients for Chinese and Russian samples are presented above and below the table diagonal line, respectively. ^*^ indicates significance at the 95% level, ^**^ indicates significance at the 99% level.

Regarding the predictive effect of emotional intelligence on physical activity, regression analysis yielded a significant model for the Chinese sample, *R*^2^ = 0.172, *F*_(4, 276)_ = 14.302, *p* < 0.001, indicating that emotional intelligence explained 17.2% of the variance in physical activity. Specifically, emotion utilization was found to be a significant predictor. In the sample of Russian students, regression analysis also yielded a significant model, *R*^2^ = 0.108, *F*_(4, 188)_ = 5.718, *p* < 0.001, indicating that emotional intelligence explained 10.8% of the variance in physical activity. Specifically, emotion utilization was found to be a significant predictor. The values of tolerance and VIF were within acceptable range. The result is presented in Table 2.

**Table 2:** Regression model results of emotional intelligence on physical activity.

**Constant**	**Unstandardized coefficients**	**Standardized coefficients**	**T value**	***P* value**	**Tolerance**	**VIF**
**Chinese**						
Constant	9.238		4.148	0.000		
SEA	−0.060	−0.009	−0.082	0.935	0.275	3.642
OEA	0.441	0.065	0.631	0.528	0.281	3.554
UOE	1.716	0.250	2.260	0.025	0.245	4.077
ROE	0.864	0.138	1.519	0.130	0.365	2.742
**Russian**						
Constant	15.631		4.977	0.000		
SEA	−0.129	−0.020	−0.188	0.851	0.421	2.375
OEA	0.001	0.000	0.002	0.998	0.761	1.314
UOE	2.623	0.355	3.931	0.000	0.582	1.718
ROE	−0.177	−0.028	−0.288	0.774	0.491	2.038

SEA, self-emotion appraisal; OEM, other's emotion appraisal; UOE, use of emotion; ROE, regulation of emotion; VIF, variance inflation factor.

To test hypothesis 3, we studied the prognostic influence of emotional intelligence on autonomous motivation, which is the most significant from the perspective of self-determined human behavior. For this purpose, we ran regression models to test such effects. The results are presented in Table 3. For the model tested in the sample of Chinese students, the overall regression model was statistically significant (*R*^2^ = 0.426, *F*_(4, 276)_ = 51.180, *p* < 0.001), indicating that emotional intelligence explained 42.6% of the variance in autonomous motivation. It was found that emotion self-appraisal and emotion regulation significantly predicted autonomous motivation, and the effect of emotion utilization on autonomous motivation was close to significance. Tolerance and VIF values were in the acceptable range.

**Table 3:** Regression model results of motivation on physical activity.

**Constant**	**Unstandardized coefficients**	**Standardized coefficients**	**T value**	***P* value**	**Tolerance**	**VIF**
**Chinese**						
Constant	11.885		4.633	0.000		
AUM	0.521	0.209	2.200	0.029	0.344	2.910
CNM	0.645	0.187	1.844	0.066	0.302	3.309
AM	−0.739	−0.152	−2.387	0.018	0.764	1.310
**Russian**						
Constant	26.169		10.792	0.000		
AUM	0.369	0.205	1.166	0.246	0.298	3.351
CNM	−0.216	−0.084	−0.454	0.651	0.267	3.745
AM	0.603	0.101	0.954	0.342	0.819	1.221

AUM, autonomous motivation; CNM, controlled motivation; AM, amotivation; VIF, variance inflation factor.

## Discussion

The main vector of research during considering the physical activity of students is associated with the substantiation of factors contributing to the increase of autonomous sports motivation. This study examined the relationship between emotional intelligence, intrinsic motivation and physical activity among student-athletes studying at institutes of physical education in China and Russia. The results of the study have revealed that Chinese students had higher scores on all components of emotional intelligence, which confirmed the hypothesis about the role of culture as a predictor of the development of emotional intelligence.

We can partially link the data we have obtained with other studies (Lane et al., 2009), which have proven that emotional intelligence positively correlates with positive emotions and with the use of psychological skills, and negatively with negative emotions.

A higher level of emotional intelligence of Chinese student-athletes is provided not only by positive emotions, such as those of Russian students. But mostly due to the ability to regulate negative emotions, which is reflected in the Chinese proverb “Emotions are not on the face,” as well as due to the possession of the techniques of “taichi,” “qigong” and other psychological skills that students at physical education universities in China master (Jiao et al., 2021).

According to the results of the two-dimensional correlation analysis, it has been revealed that in both samples, indicators of emotional intelligence have a higher correlation with autonomous motivation. Our data coincide with the results of other studies, which revealed the relationship between emotional intelligence and autonomous motivation (Mercader-Rubio et al., 2023).

We can note a certain dynamic in the relationship between emotional intelligence and sports motivation. The greatest relationship was found with autonomous motivation, the relationship with controlled motivation is significantly lower, and there is a very weak relationship or lack thereof with autonomous motivation. We have not found an explanation of the obtained data in the literature. Therefore, we believe that autonomous motivation, in which the main focus is on the effective performance of activities, is significant for the athlete himself and requires high self-control, which is provided only through conscious emotion management. Therefore, the importance of the contribution of emotional intelligence to the successful performance of activities is quite high, which was recorded in our study. It can be noted that a similar rationale for this relationship is presented in other studies (Mercader-Rubio et al., 2023). Studies have noted that emotional intelligence affects increased self-control (attention) (Ubago-Jiménez et al., 2019), wellbeing (García-Mas et al., 2021) and self-efficacy (Wang et al., 2020).

With controlled motivation, the main focus is not on the activity, but on the external result, which is associated with uncertainty, stress, pressure, while managing the emotional state does not fully ensure the achievement of the result, which reduces the contribution of emotional intelligence to achieving the goal. With sports amotivation, the focus of attention is not related to activity, so the need to manage emotions is reduced to zero.

The data we have obtained from other studies indicate that there is no connection between a low level of emotional intelligence with motivation and physical activity (Mazhar et al., 2021), a high relationship between a high level of emotional intelligence with autonomous motivation and physical activity (Castro-Sánchez et al., 2018). The predictive effect of emotional intelligence on intrinsic motivation and physical activity showed that in the Chinese sample, emotion self-assessment and emotion regulation largely predicted autonomous motivation, and the effect of using emotions on autonomous motivation was close to significant.

In the sample of Russian students, the indicators of emotional intelligence were significantly lower than in the Chinese. Therefore, in the Russian sample, only the use of emotions reliably predicts autonomous motivation. The obtained data indicate that the emotional intelligence of Russian student-athletes has a more functional role associated with ensuring the success of their activities, while for Chinese student–athletes, the value of emotions is important itself and a high ability to manage their emotions is also essential. It is important to note that in the study of the emotional intelligence of Spanish student-athletes, the lowest values among the indicators of emotional intelligence are associated with the use of emotions, while, as the authors note, they are more typical for women than for men (Castro-Sánchez et al., 2018). In our study, this conclusion is similar for Chinese student–athletes and it is not confirmed for Russian student–athletes.

Higher values of the level of emotion regulation in the Chinese sample confirm our hypothesis about the role of culture, but also indicate that a high ability to regulate emotions is a prerequisite for higher self-control of student-athletes over themselves, over managing activities and enjoying activities. But it is exactly this ability of emotional intelligence that is not only related to sports practice, but requires its conscious development. This point of view is consistent with the position of Mercader-Rubio et al. (2023). It is apparent that in sports psychology the techniques of self-regulation have always been considered to be an important component in the psychological preparation of athletes, but our study proves the influence of emotional intelligence on autonomous motivation.

Of course, in sports psychology, self-regulation techniques have always been considered an important component in the psychological preparation of athletes, but our study proves that there is the contribution of emotional intelligence as a predictor of autonomous motivation and physical activity. Moreover, the solution to this problem is not provided only by the inclusion of students in sports or physical activity, but requires the development of a strategy for the improvement of their psychological skills.

## Limitations

It should be noted that the research contains a number of restrictions which are related to the difference in social-demographic characteristics of participants from China and Russia, and first of all it relates to the number of participants. As the restriction should be considered that fact that the research was conducted online and included respondents from only one university of each country. Perception of the questions by the participants could have cultural differences, especially in the estimation of emotional intelligence. The obtained data requires further verification. Increase in the number of participants from different countries will allow to conduct a deeper analysis and interpretation of culture impact on emotional intelligence and motivation to engage in physical activity.

## Conclusion

In general, we can say that the study has allowed us to obtain new data that, on the one hand, reveal the role of culture as a factor determining the development of emotional intelligence, and on the other hand, the significant role of emotional intelligence as a predicate of autonomous motivation.

It is proved that the higher level of emotional intelligence in Chinese sample has the higher demonstrated relationship with autonomous motivation and the level of physical activity than Russian sample. In Chinese sample the most significant contribution in autonomous motivation and the level of physical activity makes such indicators as self-esteem and regulation of emotions, while in Russian selection only the indicator “using emotions” does.

Further research, in our opinion, should be aimed at taking into account the cultural context and normative forms of emotion regulation, primarily negative emotions, which can significantly reduce the development of emotional intelligence, and therefore affect autonomous motivation and physical activity levels.

## Data availability statement

The original contributions presented in the study are included in the article/supplementary material, further inquiries can be directed to the corresponding author.

## Ethics statement

The studies involving humans were approved by Ethics Committee of Ural Federal University. The studies were conducted in accordance with the local legislation and institutional requirements. The participants provided their written informed consent to participate in this study.

## Author contributions

LR: Investigation, Methodology, Writing – original draft, Writing – review & editing. TZ: Data curation, Investigation, Software, Validation, Writing – original draft, Writing – review & editing. AG-M: Conceptualization, Formal analysis, Methodology, Project administration, Supervision, Writing – original draft, Writing – review & editing.
